# De novo subtype and strain identification of botulinum neurotoxin type B through toxin proteomics

**DOI:** 10.1007/s00216-012-5767-3

**Published:** 2012-03-07

**Authors:** Suzanne R. Kalb, Jakub Baudys, Jon C. Rees, Theresa J. Smith, Leonard A. Smith, Charles H. Helma, Karen Hill, Skadi Kull, Sebastian Kirchner, Martin B. Dorner, Brigitte G. Dorner, James L. Pirkle, John R. Barr

**Affiliations:** 1 Centers for Disease Control and Prevention, National Center for Environmental Health, Division of Laboratory Sciences, 4770 Buford Hwy, N.E., Atlanta, GA 30341 USA; 2 Integrated Toxicology, United States Army Medical Research Institute of Infectious Diseases (USAMRIID), Fort Detrick, MD 21702 USA; 3 Office of the Chief Scientist, Medical Research and Materiel Command (MRMC), Fort Detrick, MD 21702 USA; 4 Bioscience Division Los Alamos National Laboratory, Los Alamos, NM 87545 USA; 5 Robert Koch-Institut, Center for Biological Security, Microbial Toxins (ZBS3), 13353 Berlin, Germany

**Keywords:** Botulinum neurotoxin, Botulism, Mass spectrometry, Proteomics

## Abstract

Botulinum neurotoxins (BoNTs) cause the disease botulism, which can be lethal if untreated. There are seven known serotypes of BoNT, A–G, defined by their response to antisera. Many serotypes are distinguished into differing subtypes based on amino acid sequence, and many subtypes are further differentiated into toxin variants. Previous work in our laboratory described the use of a proteomics approach to distinguish subtype BoNT/A1 from BoNT/A2 where BoNT identities were confirmed after searching data against a database containing protein sequences of all known BoNT/A subtypes. We now describe here a similar approach to differentiate subtypes BoNT/B1, /B2, /B3, /B4, and /B5. Additionally, to identify new subtypes or hitherto unpublished amino acid substitutions, we created an amino acid substitution database covering every possible amino acid change. We used this database to differentiate multiple toxin variants within subtypes of BoNT/B1 and B2. More importantly, with our amino acid substitution database, we were able to identify a novel BoNT/B subtype, designated here as BoNT/B7. These techniques allow for subtype and strain level identification of both known and unknown BoNT/B rapidly with no DNA required.

**Figure:**
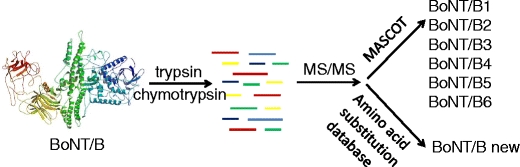
Identification of an existing or new BoNT/B can be accomplished through MS/MS analysis of digestion fragments of the protein.

Identification of an existing or new BoNT/B can be accomplished through MS/MS analysis of digestion fragments of the protein.

## Introduction

Botulism is a potentially fatal disease caused by exposure to botulinum neurotoxins (BoNTs), 150 kDa protein neurotoxins. In vivo, BoNTs halt nerve impulses by cleaving proteins necessary for acetylcholine release into the neuromuscular junction. This leads to a flaccid paralysis, which can affect respiration and may necessitate ventilator support for the patient [1]. BoNT is the most lethal toxin known with an estimated oral LD_50_ of approximately 70 μg for the average human [1]. This extreme toxicity has led, in part, to the BoNT’s current CDC designation as a category A select agent for bioterrorism, making it one of the most likely agents for bioterrorism [1]. Botulism patients receive a serotype-specific immunoglobulin products that are most effective when administered within 24 h of exposure [2]. Therefore rapid determination of exposure and identification of the BoNT serotype is an important public health goal.

There are seven different serotypes of BoNT, A–G, which are defined by their ability to be neutralized by an antiserum that was produced using a specific BoNT. Nucleotide or amino acid sequence variation within BoNTs in strains of a serotype has led to the designation of subtypes. Historically, subtypes have been defined by distinct cultural/biochemical characteristics [3], functional differences [4], or differential binding of monoclonal antibodies (mAbs) [5–7]. Within the BoNT/B serotype, there are currently six known subtypes, B1–B6 [8–11]. The B1–B6 subtypes exhibit amino acid variation of 7% or less. In addition, there is amino acid variation within a subtype, such as 1.5% within B2 or 1.7% within B4 subtypes. This variation could be a new strain (*Clostridium* organism) or toxin variant (neurotoxin protein), with some the neurotoxin of some strains having as few as a single amino acid difference, or 0.08% difference.

Identification of the subtype of BoNT is important for several reasons. First, one definition of a subtype of BoNT indicates that different subtypes of toxin might have differential binding to monoclonal antibodies, and perhaps some polyclonal antibodies as well [4,11]. This becomes important as researchers search for an alternative treatment to the currently used equine immunoglobulin approach to treat botulism. Various mAb could be proposed as immunoglobulin treatments for botulism; however, if there is differential binding of these antibodies to different subtypes, care must be taken in choosing which antibodies to use as treatment, as the antibodies might not be effective at neutralizing all subtypes of BoNT within a serotype. Secondly, identification of the BoNT subtype could be important to epidemiology and forensic investigations attempting to trace the origin of the toxin and its spread in a botulism incident. Concurrent outbreaks of botulism could be identified as originating from the identical or diverse sources based upon the subtype of toxin present.

We previously described methods to identify the serotype A subtypes BoNT/A1 or /A2 and the serotype B subtypes BoNT/B1 or /B4 using mass spectrometry [12,13]. Our methods involve a tryptic digestion of the toxin and matrix-assisted laser desorption/ionization time of flight (MALDI-TOF) or liquid chromatography–tandem mass spectrometry (LC-MS/MS) analysis of the tryptic fragments, followed by querying the data to a protein database that contains entries from the different BoNT subtypes. The subtypes BoNT/A1 and /A2 are approximately 10% different from each other, and the LC-MS/MS analysis was able to detect over half (58%) of the theoretical differences between BoNT/A1 and /A2 or 76 of the 131 residues [12]. MS/MS identification of the 76 amino acid differences provided a clear distinction of BoNT/A1 from /A2.

Currently, subtype identification is determined through DNA sequencing of the toxin’s genes [10]. Other DNA analysis techniques such as pulsed field gel electrophoresis [14], randomly amplified polymorphic DNA analysis [15], amplified fragment-length polymorphism analysis [16], *fla*A variable-region sequencing [17], multilocus sequence typing [18], multiple locus variable-number tandem repeat analysis [19], and a comparative genomic hybridization microarray [20] have also been used to differentiate strains. All of these methods rely on the presence of bacterial DNA in the sample material. However, BoNT can be present in a clarified or filtered sample in which the bacterium is absent. In such a situation, toxin subtype identification or strain characterization would be difficult, but perhaps possible, using traditional DNA-based methods [19,21] as clarified samples can contain small amounts of DNA depending on the degree of purity. If DNA is not present, then these DNA-based methods cannot be used for subtype identification.

Our method to distinguish the two BoNT/A subtypes from each other relied upon searching a database of existing proteins, which does not allow de novo identification of a new subtype or toxin variant of BoNT. De novo identification of a new subtype or variant within a BoNT subtype is just as important, if not more so, than classification of a sample as an existing subtype. In this work, we demonstrate our ability to first distinguish BoNT/B1, /B2, /B3, /B4, and /B5 subtypes from each other using mass spectrometry and the ability to distinguish multiple toxin variants within BoNT/B1 and /B2 subtypes using a amino acid substitution database that includes all possible amino acid substitutions of BoNT/B1. Finally, we describe the use of the amino acid substitution database approach to identify a novel subtype of BoNT/B, designated here as BoNT/B7. The study combines the use of mass spectrometry with the amino acid substitution database to provide a powerful tool that can be used to screen samples for new BoNT/B toxin variants or subtypes with no DNA needed.

## Experimental procedures

### Materials

BoNT is highly toxic and requires appropriate safety measures. All neurotoxins were handled in a class 2 biosafety cabinet equipped with HEPA filters. Commercially purified BoNT/B1 complex toxin was purchased (Metabiologics, Madison, WI, USA). Dynabeads® Protein G were purchased (Invitrogen, Carlsbad, CA, USA) at 1.3 g/cm^3^ in phosphate-buffered saline (PBS), pH 7.4, containing 0.1% Tween® 20 and 0.02% sodium azide. Sequencing-grade modified trypsin at 0.5 mg/mL in 50-mM acetic acid and sequencing grade chymotrypsin at 1 μg/μL in 50-mM ammonium bicarbonate was purchased (Roche, Pleasanton, CA, USA). All chemicals were from Sigma-Aldrich (St. Louis, MO, USA) except where indicated.

### Production of BoNT/B culture supernatants

Crude culture supernatants representing specific BoNT/B strains were produced by incubating subcultures of each strain for 5 days at 30–35 °C. After centrifugation, supernatants were removed and filtered through 0.22-μm filters. The filtered supernatants were tested for upper limits of toxicity using the mouse bioassay, which indicated that the toxins were all present at concentrations of ≤10 μg/mL. Information on the strains used in these studies is listed in Table 1.

**Table 1:** Strain information on culture supernatants used for this study

Sample	Strain	NCBI accession no.
B1	Okra	AB232927
B1	CDC 1656	EF028396
B1	CDC 1758	EF033127
B2	CDC 1828	EF051571
B2	Prevot 59	EF033128
B2	Prevot 25	EF033129
B3	CDC 795	EF028400
B4 (nonproteolytic)	Eklund 17B	EF051570
B5 (bivalent)	An436	EF028397
B7	Bac-04-07755	JQ354985
B7	NCTC3807	JN120760

### Neurotoxin extraction and digestion

Monoclonal antibodies 1B18 and B12.1 [22] which bind the heavy chain of the toxin obtained from the laboratory of Dr. James Marks at the University of California, San Francisco, were immobilized and crosslinked to the Dynabeads® Protein G using 30 μg of antibody diluted into 500 μL of PBS for every 100 μL of Dynabeads® Protein G. Cross-linked IgG-coated Dynabeads® were stored in PBS-Tween buffer (PBS with 0.05% Tween® 20) at 4 °C for up to 2 days. For the BoNT extraction assay, an aliquot of 20 μL of antibody-coated beads was mixed for 1 h with a solution of 200 μL of each culture supernatant and 300 μL of PBS with 0.01% Tween (PBST) buffer. After mixing for 1 h with constant agitation at room temperature, the beads were washed twice in 1 mL each of PBST and once in 100 μL of water. The beads were reconstituted in 15 μL of 50-mM ammonium bicarbonate, pH = 7.5 (tryptic buffer) and 2 μL of stock trypsin, and digested for 5 min at 52 °C^23^. Following digestion, the supernatant was then removed from the beads and 1 μL of 10% TFA was added to the supernatant. A second, independent digestion (5 min at 52 °C) was performed by reconstitution of the beads in 15 μL of tryptic buffer and addition of 1 μL of chymotrypsin diluted to 0.2 mg/mL in the tryptic buffer. After digestion, each supernatant was then removed from the beads and 1 μL of 10% TFA was added. Each digested sample was analyzed separately.

### Mass spectrometric analysis of BoNT

After digestion, 5 μL of the sample was injected onto a Waters NanoAcquity C18 5 μm UPLC trap column (180 μm ID and 2 cm long) and separated on a Waters NanoAcquity C18 1.7 μm UPLC column, 100 μm ID, and 10 cm long (Waters, Milford, MA, USA). After a trap-loading flow rate of 5 μL/min for 5 min, the peptides were eluted from the column at a flow rate of 500 nL/min using the following gradient conditions, where A is water with 0.1% formic acid and B is 100% acetonitrile with 0.1% formic acid: A = 95% and B = 5% at 0 min; A = 85% and B = 15% at 10 min; A = 65% and B = 35% at 60 min; A = 30% and B = 70% at 62 min; hold for 5 min, A = 95% and B = 5% at 70 min; hold for 10 min.

Peptides eluting from the column were introduced into a 7-Tesla linear trap (LTQ)-FT-ICR instrument (Thermo Electron, San Jose, CA) using an Advion TriVersa NanoMate nanoelectrospray ion source interface equipped with an LC coupler (Advion, Ithaca, NY, USA). The instrument was operated in data-dependent acquisition mode to automatically switch between MS and MS/MS analysis. The FT-ICR-MS was used for MS acquisition with a resolution of 25,000 from 400 to 1,600 *m*/*z*. Simultaneously, the linear ion trap was used for MS/MS analysis of the five most abundant ions in each MS scan. Automatic gain control was used to accumulate ions for FT-ICR-MS analysis with a target value of 1,000,000 and 200,000 for MS and MS/MS analysis, respectively. Collision energy of 35% was utilized in the linear ion trap to fragment the tryptic digest fragments.

### Construction of an amino acid substitution database

FASTA databases were generated using the proteolytic cleavage products of the sequence of BoNT/B1 as database entries, and single substitutions in every proteolytic cleavage product were included as an entry in the FASTA database (19 other possible amino acids per site). First, in silico digestions of BoNT/B1 based upon the known cleavage rules of trypsin and chymotrypsin, respectively, were completed and collated into cleavage product lists. Subsequently, each peptide was looped through one amino acid at a time, with each particular amino acid changed one at a time to one of the 19 other amino acids, with each changed peptide written to file as an entry in the NCBI FASTA format. Thus, using trypsin as the enzyme, the BoNT/B1 sequence generated 157 possible cleaved peptides, and with the change of one amino acid at a time, the trypsin FASTA database contained 23,899 theoretical sequences. Using chymotrypsin as the proteolytic enzyme, the algorithm generated 86 possible cleaved peptides, with 24,033 theoretical sequences. All machine code to generate the two FASTA databases was written in-house using Microsoft Visual C# 2008.

### Searching of data against databases

Peak lists for all MS/MS spectra were extracted using Mascot Distiller (Matrix Science Inc., Boston, MA, USA, version 2.2.0) and merged together using Mascot Daemon (Matrix Science Inc, Boston, MA, USA, version 2.2.2). Database searches were conducted using Mascot (Matrix Science Inc, Boston, MA, USA, version 2.2.0) against a database generated by extracting entries from the NCBI non-redundant database using “Clostridia” as the extraction parameter. Mascot parameters were applied as follows for standard protein database searching: nine maximum missed cleavages, non-specific enzyme, peptides with up to +4 charge, MS1 tolerance of 20 ppm and MS/MS tolerance of 0.8 Da, and carbamidomethyl, deamidation, and oxidation modifications allowed. Mascot parameters were applied as follows for amino acid substitution database searching: nine maximum missed cleavages, semi-tryptic (K and R) and semi-chymotryptic enzyme selectivity, peptides with up to +4 charge, MS1 tolerance of 20 ppm and MS/MS tolerance of 0.5 Da, semi-specific searches included one non-specific cleavage site located on either the N or C terminus of the peptide. MS/MS-based peptide and protein identifications were validated using Scaffold (version Scaffold_2_00_06, Proteome Software Inc., Portland, OR, USA). Peptide identifications were accepted if they could be established at greater than 95.0% probability as specified by the Peptide Prophet algorithm. Protein identifications were accepted if they could be established at greater than 99.0% probability as specified by the Protein Prophet algorithm, and if they contained at least three peptides with unique amino acid sequences.

### DNA sequencing and phylogenetic analysis

Strains were grown anaerobically in tryptone–peptone–glucose–yeast broth and DNA was isolated as reported [23]. The *bont/b* gene was amplified and subjected to Sanger sequencing. DNA alignment of the obtained full-length sequences of the *bont/b* genes with already published sequences followed by phylogenetic analysis and construction of the dendrogram was carried out as described previously [10].

## Results

Peptides derived from the tryptic and chymotryptic digestion of BoNT/B1 Okra, B2 Prevot 25, BoNT/B3 CDC 795, BoNT/B4 Eklund 17B, and BoNT/B5 An436 were analyzed by MS/MS with an LTQ-FT-ICR mass spectrometer and the data were compared against a standard protein database. All five BoNT proteins were correctly identified as BoNT/B1, B2, B3, B4, or B5 despite the high level of sequence similarity (as high as 98.4%). Table 2 is a list of peptides from the digests of these neurotoxins which were identified by MS/MS and are unique for each subtype. The overall percent coverage for each of the proteins was 76%, 76%, 66%, 75%, and 74%, respectively. Because these listed peptides are unique for each of the subtypes, they serve as biomarkers that identify each of the different BoNT/B subtypes.

**Table 2:** Peptides from the digests of BoNT/B1–/B5 which are unique for each subtype and were identified by MS/MS

Subtype	Peptide sequence
BoNT/B1 (Okra)	^384^NLLDNEIYTIEEGFNISDK**D**MEK^406^
	^595^QIV**N**DFVIEANK^606^
	^692^W**S**DMYGLIVAQWLSTVNTQFYTIK^715^
	^721^ALNYQAQALEEIIKY**R** ^736^
	^759^LNEGINQAIDNINNFIN**G**CSVSYLMK^784^
	^833^TI**M**PFDLS**I**Y^842^
	^843^TN**D**TILIE**M**FNK^854^
	^869^Y**K**DNNLIDLSGY^880^
	^1003^FVTITNNL**N**NAK^1014^
	^1021^LESN**T**DIKDIR^1031^
	^1057^YFSIFNTELSQSNIEE**R** ^1073^
	^1242^FYESGIVF**E**EYK^1253^
BoNT/B2 (Prevot 25)	^1029^ **N**IGEVIANGEIIFK^1042^
	^1162^KEDYIYLDFFNSN**R** ^1175^
	^1179^VYAYKDFKEEE**K** ^1190^
	^1242^FYESGIVLKDYK**N**YF^1256^
BoNT/B3 (CDC 795)	^255^FFMQSTADIQAEELYTFGGQDP**R** ^277^
	^884^VEVY**N**GVELNDKNQFK^899^
	^910^VTQNQ**D**IIF^918^
BoNT/B4 (Eklund 17B)	^254^KFFMQSTD**T**IQAEELYTFGGQDPSIISPSTDK^285^
	^329^FVEDSEGKYSIDVESF**N**K^346^
	^350^SLMFGFTE**I**NIAENYK^365^
	^384^NLLDNEIYTIEEGFNISDKNM**G**K^406^
	^441^ **V**PGICIDVDNE**N**LFFIADK^459^
	^472^ **V**EYNTQNNYI**G**NDFPINELILDTDLISK^499^
	^529^ **V**FTDENTIFQYLY^541^
	^542^SQTFPL**N**IR^550^
	^623^IGLALNVG**D**ETAK^635^
	^737^YNIYSE**E**EKSNINI**N**FNDINSK^758^
	^759^LNDGINQA**M**DNINDFINECSVSYLMK^784^
	^814^LYLIGS**V**E**D**EK^824^
	^833^TIIPFDLSTYTNN**E**ILI**K** ^850^
	^936^Y**R**ND**D**IQNYIHNEYTIINCMK^956^
	^1015^IYING**T**LESN**M**DIK^1028^
	^1029^DIGEVIV**N**GEI**T**FK^1042^
	^1042^KLDGD**V**DRTQF^1052^
	^1057^YFSIFNT**Q**L**N**QSNIK^1071^
	^1111^L**V**KDSSVGEIL**I**R^1123^
	^1163^EDYI**H**LDF**V**NSN**E**EWR^1178^
	^1184^NFKE**Q**E**Q**K^1191^
	^1192^LFL**SI**IYDSNEFYK^1205^
	^1226^KDEESTD**D**IGLIGIHR^1241^
	^1242^FYESGVL**R** ^1249^
	^1272^ **K**SNLGCNWQFIPKDEGWTE^1290^
BoNT/B5 (An436)	^472^I**A**YNTQNNYI**D**NDFSINELILDTDLISK^499^
	^500^IELPSENTESLTDFNV**Y**VP**E**Y**K** ^521^
	^677^II**E**TI**NS**ALTK^687^
	^746^SNINIDFND**V**NSK^758^
	^794^LLDFDNTL**R** ^802^
	^833^T**S**IPFDLSTY^842^
	^855^YNS**D**ILNNIILNLR^868^
	^983^SVFFEYSI**K** ^991^
	^1032^EVIAN**D**EIIFK^1042^
	^1043^LDG**N**IDRTQFIWMK^1056^

Residues which make each peptide unique for the given subtype are bolded

Although these five BoNT proteins show as much as 98.4% identity, there are still many differences in the amino acid sequence among these toxins, and these differences can be exploited to distinguish toxins from each other by mass spectrometry. For example, there are 56 amino acids that differ between BoNT/B1 Okra and B2 Prevot 25. Upon MS examination of BoNT/B1 Okra and B2 Prevot 25, we obtained evidence for 34 of these amino acids or approximately 60% of the differing amino acid residues.

The 34 amino acid differences were identified by querying the MS/MS data against a protein database that included a protein sequence (BoNT/B2 Prevot 25) containing all 56 differing amino acids. However, protein sequences of any novel BoNT/B subtypes would not be present in the protein database and therefore, a unique new subtype of BoNT/B would simply be identified as the closest match that exists within the database. To optimize identification of novel subtypes, we developed an amino acid substitution database in which every amino acid within the BoNT/B1 Okra sequence was mutated in silico to 19 other possibilities. It is important to note that this amino acid substitution database was not intended to include all amino acid differences, as it does not account for more than one mutation within a tryptic or chymotryptic fragment, but rather suggests the presence of a new subtype or variant, and assists in the differentiation of toxins from botulism outbreaks. Although the database would certainly be more inclusive through the inclusion or more than one mutation within a tryptic or chymotryptic fragment, it would also require substantially more time to search against, thus lengthening the time frame for identification.

**Fig. 1:**
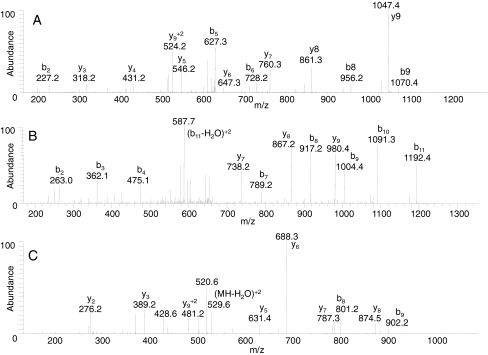
MS/MS spectra of the precursor ions at *m*/*z* 637.36 (*1A*), 671.32 (*1B*), and 538.79 (*1C*) corresponding to doubly-charged peptides IIWTL**T**DINGK (*1A*), DFVIEANKS**S**TM (*1B*), and DS**S**VGEILTR (*1C*) from the digests of BoNT/B2. The spectra were obtained by LC-MS/MS on an FT-ICR mass spectrometer, with MS analysis in the FT-ICR cell and MS/MS analyses within the LTQ portion. Residues which are set in *bold* indicate amino acid substitutions

We first tested the use of the amino acid substitution database by digesting the BoNT/B2 Prevot 25 and treating it as an unknown while searching the data in the /B1 Okra amino acid substitution database. BoNT/B1 Okra and BoNT/B2 Prevot 25 are fairly similar (95.6%) with 56 amino acids that differ. Upon querying the data from the digests of BoNT/B2 Prevot 25 against the /B1 Okra amino acid substitution database, MS/MS data were found to support the identity of ten amino acid differences; the MS/MS spectra which demonstrate the presence of some of those differences are shown in Fig. 1. The ten amino acids represent 18% (10 of 56) of the total known differences

Table 3 lists the peptides with amino acid differences that were discovered through the use of the amino acid substitution database. Figure 1a is the MS/MS of the sequence ^970^IIWTL**T**DINGK^980^ which includes the difference from I to T in position 975. In addition to the altered intact peptide ion at *m*/*z* 637.36, the mass difference between *y*_5_ at *m*/*z* 546.2 and *y*_6_ at *m*/*z* 647.3 is 101 Da, which corresponds to a threonine rather than an isoleucine. Additionally, the mass difference between *b*_5_ at *m*/*z* 627.3 and *b*_6_ at *m*/*z* 728.2 is 101 Da, which corresponds to a threonine rather than an isoleucine. This MS/MS spectrum indicates the existence of the amino acid substitution from I to T in position 975.

**Table 3:** Peptides from the digest of both BoNT/B1 and /B2 with amino acid differences that were discovered through the use of the amino acid substitution database

Amino acid position	Difference (B2 Prevot 25)	Normal (B1 Okra)
472-484	IEYNTQSNYIEN**R**	IEYNTQSNYIEN**D**
599-610	DFVIEANKS**S**TM	DFVIEANKS**N**TM
850-868	E**I**FNKYNSEILNNIILNLR	E**M**FNKYNSEILNNIILNLR
876-887	DLSGYGA**N**VEVY	DLSGYGA**K**VEVY
910-923	VTQNQNIIFNS**M**FL	VTQNQNIIFNS**V**FL
970-980	IIWTL**T**DINGK	IIWTL**I**DINGK
976-992	DINGKTKSVFFEY**S**IRE	DINGKTKSVFFEY**N**IRE
1057-1071	YFSIFNTELSQSNI**K**	YFSIFNTELSQSNI**E**
1072-1084	E**I**YKIQSYSEYLK	E**R**YKIQSYSEYLK
1114-1123	DS**S**VGEILTR	DS**P**VGEILTR

Figure 1b is the MS/MS of the sequence ^599^DFVIEANKS**S**TM^610^ with the mutation from N to S in position 608. The altered intact peptide doubly charged ion at *m*/*z* 671.32, and the mass difference of 87 Da between *b*_9_ at *m*/*z* 1,004.4 and *b*_10_ at *m*/*z* 1,091.3, demonstrate the existence of a serine rather than an asparagine. Figure 1c is the MS/MS of the sequence ^1114^DS**S**VGEILTR^1123^ which includes the mutation from P to S in position 1116. In addition to the altered intact peptide ion at *m*/*z* 538.79, the mass difference between *y*_7_ at *m*/*z* 787.3 and *y*_8_ at *m*/*z* 874.5 is 87 Da, which corresponds to a serine rather than a proline; this confirms that this peptide has an amino acid substitution in position 1116. The discovery of these ten amino acids indicates that the amino acid substitution database can be used for de novo discovery of many of the differences present in the B2 Prevot 25 sequence. It also demonstrates that a previously unknown subtype of BoNT/B could be identified as “novel” through the use of the amino acid substitution database.

We then used this approach to distinguish BoNT/B1 toxin variants, whose amino acid sequences are more similar to BoNT/B1 Okra than that of a different BoNT/B subtype, like BoNT/B2 Prevot 25. Two strains identified as producing BoNT/B1, but whose protein sequences were not entered into the protein database, were tested. The BoNT/B1 strain CDC 1656 has nine amino acid differences compared to BoNT/B1 strain Okra, and two of the nine differences were discovered through the use of the amino acid substitution database, as seen in Table 4. Strain CDC 1758 has only three amino acid differences for a variation of less than 0.3%, and one of those differences was discovered through the use of the amino acid substitution database as seen in Table 4.

**Table 4:** Locations and type of mass-spectrometric identified amino acid differences in two additional strains of BoNT/B2 as compared to BoNT/B1 Okra

B1 Okra residue	B1 CDC 1656 residue	B1 CDC 1758 residue	B1 Okra peptide identified by MS/MS	B1 CDC 1656 peptide identified by MS/MS	B1 CDC 1758 peptide identified by MS/MS
70C	70W	70C	DV**C**EYYDPDYLNTNDKK	DV**W**EYYDPDYLNTNDK	DV**C**EYYDPDYLNTNDK
1250E	1250K	1250K	FYESGIVF**E**EYK	FYESGIVF**K**EYK	FYESGIVF**K**

Two additional strains expressing BoNT/B2, with the sequence of their *bont* genes confirmed by full-length sequencing, but whose protein sequences were not entered into the protein database, were also analyzed using this approach. BoNT/B2 strain CDC 1828 has 58 amino acid differences compared to BoNT/B1 strain Okra, and, as indicated in Table 5, seven of those differences were discovered through the use of the amino acid substitution database. This corresponds to 23% of the total number of peptides which have a single amino acid difference. BoNT/B2 strain Prevot 59 has 56 amino acid differences, and as indicated in Table 5, seven of those differences were discovered by querying the amino acid substitution database, corresponding to 26% of the total number of peptides which have a single difference.

**Table 5:** Locations and type of mass spectrometric identified amino acid differences in two additional strains of BoNT/B2 as compared to BoNT/B1 Okra

B1 Okra residue	B2 CDC 1828 residue	B2 Prevot 59 residue	B2 CDC 1828 peptide identified by MS/MS	B2 Prevot 59 peptide identified by MS/MS
608N	608S	608S	DFVIEANKS**S**TM	DFVIEANKS**S**TM
851M	851I	851I		E**I**FNKYNSEILNNIILNLR
870K	870R	870R	YNSEILNNIILNLRY**R**	
883K	883N	883N	DLSGYGA**N**VEVY	
921V	921M	921M	VTQNQNIIFNS**M**FL	VTQNQNIIFNS**M**FL
975I	975T	975T	IIWTL**T**DINGK	
1031R	1031G	1031G		DI**G**EVIANGEIIFK
1071E	1071K	1071K	SIFNTELSQSNI**K**	YFSIFNTELSQSNI**K**
1073R	1073I	1073I		E**I**YKIQSYSEYLK
1116P	1116S	1116S	DS**S**VGEILTR	DS**S**VGEILTR

**Fig. 2:**
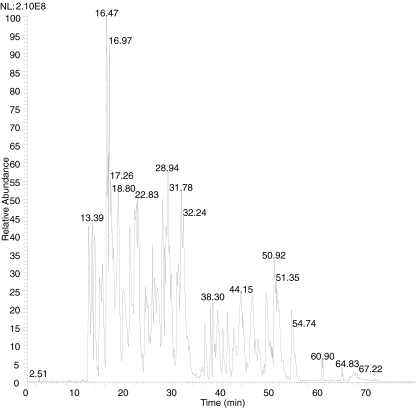
Chromatogram of the tryptic digest of a BoNT/B obtained by LC-MS on an FT-ICR mass spectrometer

**Fig. 3:**
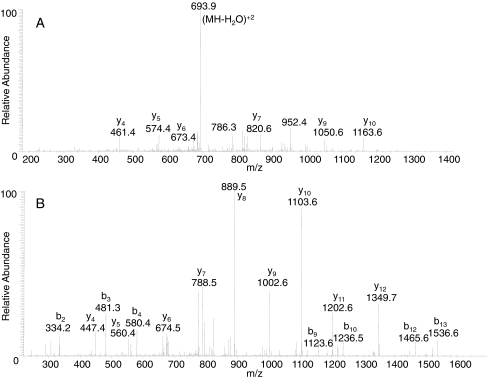
MS/MS spectra of precursor ions at *m*/*z* 702.89 (*3A*) and 841.93 (*3B*) corresponding to doubly charged peptides QI**I**DDFVIEANK (*3A*) and WFFVTITNN**L**DNAK (*3B*) from the digests of BoNT/B7 Bac-04-07755. The spectra were obtained by LC-MS/MS on an FT-ICR mass spectrometer, with MS analysis in the FT-ICR cell and MS/MS analyses within the LTQ portion. Residues which are set in *bold* indicate amino acid substitutions

As a final demonstration of our ability to identify a BoNT/B sample as “novel”, we analyzed several samples which were determined to contain BoNT/B after testing by mouse bioassay and Endopep-MS [22]. However, these samples had not yet been subjected to DNA analysis, so there was no subtype or strain identification on the samples. After digestion, MS/MS analysis, and searching of the standard protein database, one of the samples was tentatively identified as BoNT/B2 L-590 with 48% sequence coverage, with the chromatogram shown in Fig. 2. After searching the MS/MS data from this sample against the amino acid substitution database, five differences compared to BoNT/B2 L-590 were found, indicating that this protein is not BoNT/B2 L-590. Specifically, these five differences were found to be V597I/L, S746I/L, L877S, S1010I/L, and A1035V, and MS/MS evidence for some of these differences are shown in Fig. 3. Before this work, amino acid positions 597, 746, and 877 were not known to have differences in any strain or subtype of BoNT/B, so these differences are completely new for BoNT/B.

Figure 3a is the MS/MS of the sequence ^595^QI**I**DDFVIEANK^606^ which includes the mutation from V to I in position 597. In addition to the altered intact peptide ion at *m*/*z* 702.9, the mass difference between *y*_9_ at *m*/*z* 1,050.6 and *y*_10_ at *m*/*z* 1,163.6 is 113 Da, which corresponds to a leucine or isoleucine rather than a valine. Residue 1010, an I or L in this sample, is an L in some BoNT/B1 and B4, but this difference is new for any BoNT/B2 where this residue is typically a serine. Figure 3b is the MS/MS of the sequence ^1001^WFFVTITNN**L**DNAK^1014^ which includes the mutation from S to L in position 1010. The intact peptide ion at *m*/*z* 841.93 is different from *m*/*z* 828.90, which appears in all other BoNT/B2. The mass difference between *y*_4_ at *m*/*z* 447.4 and *y*_5_ at *m*/*z* 560.4 is 113 Da, which corresponds to a leucine or isoleucine. Furthermore, the mass difference between *b*_9_ at *m*/*z* 1,123.6 and *b*_10_ at *m*/*z* 1,236.5 is 113 Da, which corresponds to a leucine or isoleucine. Residue 746 is an S in all other BoNT subtypes and was not known until now to be mutated. Similarly, residue 877 is an L in all other BoNT/B subtypes. Residue 1035 is a V only in BoNT/B4 subtypes, and this difference is new for any BoNT/B2.

**Fig. 4:**
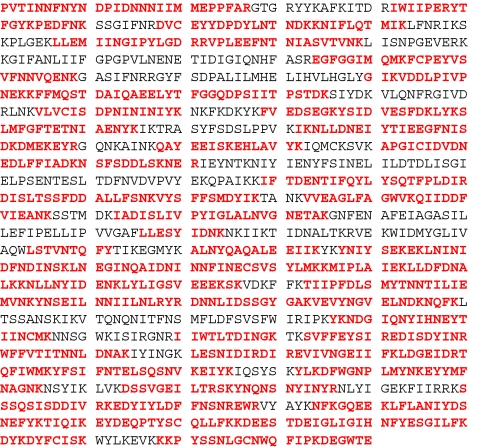
Amino acid sequence of BoNT/B7 Bac-04-07755. Residues in *red* comprise the 68% sequence coverage for which MS/MS evidence was obtained

After discovering these novel point mutations in the BoNT/B sample, the *bont/b* gene was sequenced confirming the above point mutations as well as 45 other amino acid differences, defining this sample as a new subtype of BoNT/B, BoNT/B7. After adding this new BoNT/B sequence to our standard database and searching the MS/MS data against the updated database, the top hit was the new BoNT/B7 Bac-04-07755 strain. The percent coverage increased from 48% coverage with BoNT/B2 L-590 to 68% coverage with BoNT/B7 Bac-04-07755 as seen in Fig. 4, further verifying that this is a more accurate identification of the BoNT/B in this sample.

## Discussion

Our experiments demonstrate the ability to identify the subtype (B1-B5, B7) of a BoNT/B using both the MS/MS with a standard protein database or with our amino acid substitution database. The combination of the two methods also can identify the subtype, a toxin variant within a subtype, or identify a unique subtype, not previously discovered. In previous work, we demonstrated that BoNT subtypes could be distinguished from each other by MS [12,13]. While Kull et al. used MALDI-TOF MS to differentiate between BoNT/B1 and B4, we previously analyzed the tryptic peptides of BoNT/A1 and A2 by LC-MS/MS. Although these two A subtypes are approximately 90% homologous, we demonstrated that we could exploit the 10% of the protein that was unique, to identify the toxin as BoNT/A1 or /A2. The amino acid sequences of BoNT/B1–B5 are more conserved than A1 and A2. They differ by as little as 1.6% (in the case of BoNT/B2 and /B3), while the difference between /A1 and /A2 is 10.1%. Although these five BoNT/B subtypes are very similar, they still differ by as few as 21 amino acids, which was sufficient for subtype identification through enzymatic digestion and subsequent mass spectrometric analysis.

The use of two different enzymes (trypsin and chymotrypsin) to digest the samples contributes to the high percent coverage of these proteins with a mass above 150 kDa. A high percentage of coverage is critical for novel BoNT subtype and toxin variant identification as with increasing coverage (amino acid sequence for which there is mass spectrometric evidence), there is a higher likelihood of identifying more amino acid differences. Through the use of the combined enzymatic digestions and LC-MS/MS analysis of the resultant peptides, we were able to obtain 77%, 76%, 66%, 75%, and 74% amino acid coverage of BoNT/B1, /B2, /B3, /B4, and /B5, respectively. This high sequence coverage allowed us to use mass spectrometry to distinguish each of these highly similar proteins from each other, and in the case of /B1 and /B2, to identify 34 of the 56 amino acids which differed between these two proteins. Although less than 40% of the differing residues could not be detected by mass spectrometry; nonetheless, the identification of 34 amino acid differences provides an effective method allowing for a definitive identification of BoNT/B as either the /B1 or /B2 subtype. Furthermore, these studies represent the first protein sequencing method applied to BoNT/B2, B3, B4, and /B5.

BoNT/B5 is a bivalent toxin producer, producing both BoNT/B5 and a lesser amount of BoNT/F or A. Due to the specificity of the capture antibodies, we can examine bivalent toxin strains without interference from the second toxin. The detection of two toxins in a preparation can be difficult for some other identification methods. Identification of the toxin as a /B5 through proteomic analysis might indicate that there is a second toxin present which could have an effect on botulism treatment. Additionally, the /B4 subtype is associated with nonproteolytic BoNT/B strains. These strains are not always reliably detected using other methods, but the proteomic technique discussed here can easily identify BoNT/B4.

If the amino acid sequence of BoNT/B2 Prevot 25 had not been in the protein database, as would be expected for an unknown subtype or toxin variant of BoNT/B, we still would have identified the protein as discrete from that of BoNT/B1 Okra through the use of the amino acid substitution database. The creation of the database allowed the “de novo” identification of ten amino acid differences. Although not all of the theoretical differences were discovered using this method, there were enough differences to assign the identity of BoNT/B2 as similar to BoNT/B1, but not as BoNT/B1—a more precise identification than the de facto assignment of BoNT/B1. Since our goal is not to identify all possible amino acid differences, but identify a BoNT protein as an existing subtype or a novel toxin variant and to identify differences in toxins from ongoing botulism outbreaks; these results demonstrate that this goal can be achieved through the use of this technique.

To test the ability of the method to detect differences within subtypes (<1.4% amino acid variation which is ~21 amino acids), we obtained two BoNT/B1 samples whose amino acid sequences were not in the protein database, digested those proteins, and analyzed the resultant fragments by LC-MS/MS. Searching the data against an amino acid substitution database of BoNT/B1 Okra allowed for identification of two of nine possible differences in one strain and one of three possible differences in another strain which is sufficient to indicate differences in outbreak samples.

The identities of residues 70 and 1250 are critical for identification of these strains as three separate proteins. According to data listed in Table 4, BoNT/B1 Okra contains 70C and 1250E, BoNT/B1 CDC1656 contains 70W and 1250K, and BoNT/B1 CDC 1758 contains 70C and 1250K. Because each of these samples has a unique combination of identities of residues 70 and 1250, these three proteins can be identified as three separate protein identities. Again, although not all differences were discovered through this technique, the goal of strain differentiation of BoNT/B1 by mass spectrometry was achieved, as these three samples were identified as having very similar yet distinctly unique protein sequences. Additionally, this work reports the first protein comparison of multiple strains of the same BoNT subtype by MS techniques.

Strain variance exists in most of the BoNT subtypes, and BoNT/B2 is not an exception. We obtained two BoNT/B2 samples whose sequences were not in the protein database, digested those samples, and the resultant peptides were analyzed by LC-MS/MS. The data were then queried against the amino acid substitution database generated from the BoNT/B1 Okra sequence. Seven to ten of the possible 56–58 amino acid differences were discovered using this technique. Note that searching the data through the amino acid substitution database yielded one to two amino acid differences for toxin variants (BoNT/B1) and seven to ten amino acid differences for subtype (BoNT/B2) differences. Therefore, this technique could allow for a tentative identification of a novel protein or a new BoNT/B subtype or toxin variant, depending on the number of amino acid differences discovered through this technique.

In fact, the number of amino acid differences was critical toward our tentative identification of the BoNT/B7 sample as a new subtype rather than a new BoNT/B2 strain/toxin variant, as this sample contained five novel amino acid differences, or greater than the number of one to two that we saw for strain differentiation. BoNT/B7 strain Bac-04-07755 originates from a recent infant botulism case in New York. Interestingly, the identical novel subtype BoNT/B7 was independently discovered by BGD and co-workers by “classical” DNA sequencing of strain NCTC 3807 (National Collection of Type Cultures, UK). Strain NCTC 3807 was originally isolated in 1921 from soil from the Blue Ridge Mountains of Virginia and later deposited into the NCTC strain collection [24], meaning that this “old” strain is still circulating within the USA. Noteworthy, this new subtype BoNT/B7 has been reported to differ from other BoNT/B molecules, as the toxin was not recognized by a mAb in a specific ELISA [5], again justifying that this toxin should be named a novel BoNT/B subtype.

**Fig. 5:**
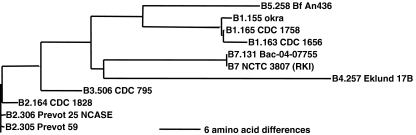
Dendrogram of proteins used in this study. Because the *horizontal line* represents the distance for six amino acids, the closer the subtypes in distance the fewer differences in amino acid between them

Indeed, DNA sequencing and subsequent phylogenetic analysis [10] showed that strains Bac-04-07755 and NCTC 3807 form a separate cluster, here designated as BoNT/B7, adjacent to the BoNT/B4 cluster as seen in the dendrogram in Fig. 5.

Although the dendrogram shows that its closest relative is BoNT/B4, searching the MS/MS data of BoNT/B7 against the known protein database yielded BoNT/B2 as the most closely related protein. This is simply because unlike DNA sequencing, the mass spectrometer did not identify 100% of the protein and as a result could not be used solely to determine evolutionary relatedness. The portion of the protein identified by mass spectrometry was most similar to BoNT/B2. This is not problematic as the goal of this work is not to identify the closest neighbor of the toxin, but rather to identify the toxin as a new subtype or toxin variant.

Because our technique identified single-point mutations within a peptide, conservative data interpretation is a key component of correct identification. Mass spectrometry data do not always provide evidence for every amino acid within a peptide, so a mass spectrum could be misinterpreted and provide misleading results. Specifically, it is not enough for the intact peptide mass and the fragmentation pattern to match a new peptide; it is critical that product ions be obtained to prove the identity of the single mutated residue. As an example, it was initially suspected that the identity of a peptide at *m*/*z* 888.94 was the doubly charged peptide YFSIFNTELSQS**V**IE, with a single amino acid substitution from the sequence of YFSIFNTELSQS**N**IE. Product ions from y_4_ to y_11_ appeared to confirm this finding. However, with the absence of the y_2_ and y_3_ or b_12_ and b_13_ ions, this finding was not confirmed. After PCR sequencing, this peptide was found to have the identity of YFSIFNTELSQN**VK**, or two differences within the peptide. This peptide also produced an *m*/*z* of 888.94 and the same y_4_ to y_11_ product ions as produced with the peptide sequence YFSIFNTELSQS**V**IE. Because our amino acid substitution database only supplied single-point mutations within a peptide, the correct possibility was not considered, and a less conservative data analysis would have produced a misleading result.

By examining the tryptic and chymotryptic digests of BoNT/B by mass spectrometry, a toxin can be first identified as BoNT/B and then further identified as subtype /B1, /B2, /B3, /B4, or /B5 similar to previous experiments which identified BoNT/A as A1 or A2 [12]. Additionally, we have demonstrated that novel BoNT/B subtypes or toxin variants could be reported using the point mutation search approach, rather than incorrectly identified as the most similar protein match in the database of known protein sequences. Although the use of an amino acid substitution database does not identify all of the amino acid differences present in a novel toxin variant or subtype of BoNT/B, it identifies enough of the amino acid differences to enable classification of a toxin as a new BoNT/B subtype or even a new toxin variant. Such information is especially important for forensics and epidemiological purposes in order to identify the possible source of an outbreak or the spread of BoNT-producing *Clostridium* species.

Because this information can be obtained without DNA, it is possible to identify BoNT/B to or below the subtype level using protein-based analysis. This technique is, to our knowledge, the only protein-based technique for BoNT subtyping and toxin variant identification, providing a relatively rapid and accurate protein subtyping tool for analysis of botulism samples. Additionally, such information can be obtained in a few days rather than a few weeks, making it a valuable tool for epidemiologists who are tracking botulism outbreaks on a real-time basis. Currently, the amino acid substitution database is searched manually, and one future goal is to automate this process. We are optimistic that automation of data searching would allow for novel BoNT subtype and toxin variant identification within 1 day as sample preparation and data acquisition occurs in only a few hours.
